# Functional Integration and Segregation in a Multilayer Network Model of Patients with Schizophrenia

**DOI:** 10.3390/brainsci12030368

**Published:** 2022-03-10

**Authors:** Jing Wei, Xiaoyue Wang, Xiaohong Cui, Bin Wang, Jiayue Xue, Yan Niu, Qianshan Wang, Arezo Osmani, Jie Xiang

**Affiliations:** 1 College of Information and Computer, Taiyuan University of Technology, Taiyuan 030024, China; 20141032@sxufe.edu.cn (J.W.); wangxiaoyue0294@tyut.edu.cn (X.W.); cuixiaohong@tyut.edu.cn (X.C.); wangbin01@tyut.edu.cn (B.W.); xuejiayue0062@link.tyut.edu.cn (J.X.); niuyan@tyut.edu.cn (Y.N.); wangqianshan0060@link.tyut.edu.cn (Q.W.); osmaniarezo@gmail.com (A.O.); 2 School of Information, Shanxi University of Finance and Economics, Taiyuan 030024, China

**Keywords:** schizophrenia, brain network, frequency, multilayer network, rs-fMRI

## Abstract

Research has shown that abnormal brain networks in patients with schizophrenia appear at different frequencies, but the relationship between these different frequencies is unclear. Therefore, it is necessary to use a multilayer network model to evaluate the integration of information from different frequency bands. To explore the mechanism of integration and separation in the multilayer network of schizophrenia, we constructed multilayer frequency brain network models in 50 patients with schizophrenia and 69 healthy subjects, and the entropy of the multiplex degree (EMD) and multilayer clustering coefficient (MCC) were calculated. The results showed that the ability to integrate and separate information in the multilayer network of patients was significantly higher than that of normal people. This difference was mainly reflected in the default mode network, sensorimotor network, subcortical network, and visual network. Among them, the subcortical network was different in both MCC and EMD outcomes. Furthermore, differences were found in the posterior cingulate gyrus, hippocampus, amygdala, putamen, pallidum, and thalamus. The thalamus and posterior cingulate gyrus were associated with the patient’s symptom scores. Our results showed that the cross-frequency interaction ability of patients with schizophrenia was significantly enhanced, among which the subcortical network was the most active. This interaction may serve as a compensation mechanism for intralayer dysfunction.

## 1. Introduction

Schizophrenia is a common chronic and disabling mental disorder, although its neural basis remains unclear. Relevant studies have shown that schizophrenia is a typical complex mental disorder with abnormal connections in the whole brain, and its underlying neuropathology is related to the abnormal functional coordination of multiple brain regions in patients [1].

Brain activity is frequency-specific, and different physiological activities produce frequency-specific signals [2,3]. Recent research has found that brain network differences in schizophrenia occur at different frequencies [4]. Researchers also found that schizophrenia patients showed a wide range of frequency band-specific differences in low-frequency amplitude and regional consistency [5]. However, previous studies have shown that cross-frequency coupling was used the deployment of spatial attention during visuomotor tasks [6,7]. Moran et al. also pointed out that the study of intraband or interband interactions at specific frequencies plays an important role in the study of schizophrenia [8]. Therefore, different frequency bands cannot be regarded simply as single entities. Ignoring the interaction between frequency bands may lead to the loss of some important information. An effective way to solve this problem is to use a multilayer network, which can integrate the interdependencies from within and between layers simultaneously [9,10].

Research has demonstrated that, compared with healthy subjects, the brain network topological characteristics of patients with schizophrenia was disordered [11]. This indicated brain functional network tissue dysfunction in patients. Functional segregation and integration are two major principles of human brain organization. An optimal brain needs to strike a suitable balance between local specialization and global integration of activities, which is well reflected by the small-world attribute [12]. Several recent studies showed impairment in the state-related characteristics of both functional integration and segregation of brain networks in patients with schizophrenia [13,14]. Segregation studies report hyper-segregated features in the posterior cortical and subcortical (striatum) brain regions [15,16]. Other studies reported on the default mode network (DMN) to whole-brain connectivity as a predictive biomarker [17,18,19]. Another study reported that, compared with healthy controls, patients with schizophrenia at baseline showed lower clustering coefficient and local network efficiency [13]. However, it is not clear how these two indicators of the brains of patients with schizophrenia can be quantified in a multilayer network. Therefore, we researched the integration and segregation of multilayer networks.

**Figure 1:**
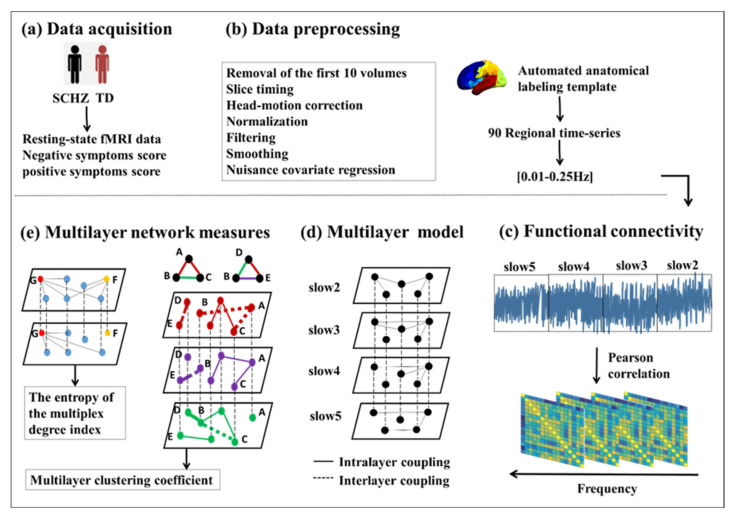
Schematic overview of the analysis strategy. (**a**) The resting-state fMRI data of schizophrenia (SCHZ) and typical development (TD) were obtained. At the same time, the negative and positive symptom scores of the patients were obtained. (**b**) This panel describes the preprocessing procedure of data, and brain signals of 0.01–0.25 Hz were obtained. (**c**) The brain signals between 0.01 and 0.25 Hz were decomposed into four frequency bands (slow2–slow5), and Pearson correlation was used to establish intralayer functional connections. (**d**) The corresponding nodes are considered connected between different layers. In this way, a multilayer frequency network model was constructed. (**e**) Multilayer network measures were calculated.

Here, we explored the dynamic functional characteristics of the whole brain of patients with schizophrenia from a multifrequency perspective. A multilayer network model was first constructed; then, we evaluated the integration and segregation characteristics of the multilayer frequency brain network in patients with schizophrenia. In addition, the dynamic role of resting-state networks (RSNs) in multilayer networks was analyzed to further evaluate the correlation between the properties of multilayer networks and patients’ clinical symptoms. The analysis strategy flow of this study is shown in Figure 1.

## 2. Materials and Methods

### 2.1. Participants and Data Acquisition

The resting-state fMRI data used in this study were acquired from the University of California LA Consortium for Neuropsychiatric Phenomics study, which was approved by the UCLA Institutional Review Board. The experiments were undertaken with the understanding and written consent of each subject. This dataset includes 119 subjects, 50 schizophrenia patients, and 69 healthy people with matching ages and sex. Table 1 shows the demographic information of the participants. Positive symptom severity was assessed by the Scale for Assessment for Positive Symptoms (SAPS) [20]. Negative symptom severity was assessed by the Scale for the Assessment of Negative Symptoms (SANS) [21].

Neuroimaging data were obtained on a 3T Siemens Trio scanner. During the process of data acquisition, all subjects were required to keep their eyes open, to remain relaxed, and to try to avoid mental activities. The fMRI data were collected with T2*-weighted echo-planar imaging (EPI). The scanning parameters were as follows: slice thickness = 4 mm, slices = 34, repetition time (TR) = 2 s, echo time (TE) = 30 ms, flip angle = 90°, field of view (FOV) = 192 mm, and matrix = 64 × 64. For the structural scan, the equipment parameters for the acquisition of T1-weighted magnetization-prepared rapid gradient echo (MP-RAGE) sagittal images were as follows: slice thickness = 1 mm, slices = 176, TR = 1.9 s, TE = 2.26 ms, matrix = 256 × 256, and FOV = 250 mm.

### 2.2. Data Preprocessing

The resting-state fMRI data were obtained using the Data Processing Assistant for Resting-State fMRI (DPARSF) toolbox [22] and Statistical Parametric Mapping (SPM12) [23]. Briefly, data from the first 10 time points were removed, and slice timing correction was performed. Then, the images of all subjects were realigned for head movement, which did not exceed 2.0 mm of displacement or 2.0° of rotation in any direction. The image space was standardized to the Montreal Institute of Neurology (MNI) head anatomy template and resampled with 3 mm × 3 mm × 3 mm voxels. The linear trends of time series were removed, and the effect of nuisance covariates was removed by signal regression using the global signal, the motion parameters, the cerebrospinal fluid (CSF), and white matter (WM) signals. Subsequently, 0.01–0.027 Hz (slow5), 0.027–0.073 Hz (slow4), 0.073–0.198 Hz (slow3), 0.198–0.25 Hz (slow2), and 0.01–0.25 (full-frequency) bandpass filters were applied to the time series of each voxel [24]. Finally, a Gaussian filter with a full width at half maximum (FWHM) of 6 mm was used to smooth the image.

### 2.3. Single-Layer Network Construction

The cerebral cortex was divided into 90 brain regions using an automated anatomical labeling (AAL) template [25]. Pearson correlation was used to calculate the functional connections between brain regions to build single-layer networks for five frequency bands. In the experiment, the correlation coefficient matrix was binarized by setting a threshold value so that all resultant networks have comparable topological structures with the same number of edges. We used the equal interval sparsity threshold range (ranging from 0.1 to 0.4 with a partition interval of 0.05) The sparsity range used in this paper was 0.1–0.4 with an interval of 0.05. The brain functional network of all subjects was constructed under all sparsity levels.

### 2.4. Multilayer Network Construction

For multilayer networks, a common representation is the supra-adjacency matrix [26,27]. A multilayer network with M layers can be expressed as follows:

(1) $$\left[\begin{array}{ccc} A^{\left[1\right]} & \cdots & I_{N}\\ \vdots & \ddots & \vdots \\ I_{N} & \cdots & A^{\left[M\right]} \end{array}\right],$$

where $A^{\left[\alpha \right]}\;$ is an *N* × *N* (*N* = 90) adjacency matrix of the four frequency bands described above, representing the interregional connectivity, and $a_{ij}^{\left[\alpha \right]}=1$ if node i and node j are connected through a link on layer α. *M* is the number of layers, and M=4. These constitute the four independent layers of the multilayer network model. Adjacency matrices $I_{N}$ were generated by connecting different frequency bands, which represent the regional connectivity between different layers. $I_{N}$ is the *N*-dimensional identity matrix, which represents the interlayer interaction of the multilayer network model.

### 2.5. Network Measures

After the brain network was constructed, the network attributes were calculated for each selected threshold. Because different threshold values could result in different brain functional networks, the attribute values of each network were also quite different. Therefore, we calculated the area under the curve (AUC) of each node attribute under different thresholds. The AUC value was taken as the node attribute measurement in this study, thus representing the overall characteristics of the index in the selected threshold space.

### 2.6. Single-Layer Network Measures of Segregation and Integration

For single-layer networks, we used the clustering coefficient (C_p_) and the local network efficiency (E_loc_) to measure segregation and integration. The computations of C_p_ and E_loc_ were performed using MATLAB codes termed GRETNA [28] (http://www.nitrc.org/projects/gretna/ (accessed on 3 October 2021)).

### 2.7. Multilayer Network Segregation

The clustering coefficient in brain networks is an indicator that can be used to characterize the local connectivity of networks and that reflects the functional differentiation mechanism of the cerebral cortex. In general, clustering coefficients are defined in two ways. The first is to quantify the likelihood that two neighbors of node i will connect to each other. For node i, its immediate neighbor node is found in set k; then, the clustering coefficient is set equal to the number of edges in the network composed of k divided by the number of possible edges in the k set. The second calculation method, similar to network transitivity, uses the ratio of closed triples to connected triples. Since each layer of a multilayer network can be regarded as a single-layer network, the definition of the network clustering coefficient and network transitivity can be used to describe the richness of triangles in each layer.

**Figure 2:**
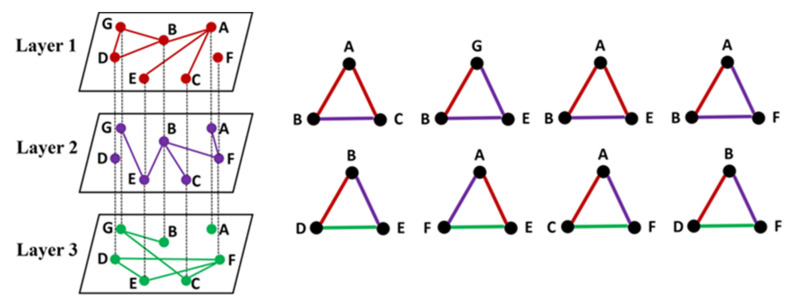
The formation of triangles in a multilayer network. On the right side of the figure are two rows of triangles, each representing one case. For the first case, the sides of the triangle are made up of two layers. Taking nodes A, B, and C as examples, node B and node C in layer 1 belong to the neighbor nodes of node A, but there is no connecting edge between node B and node C. The connection edge between them is detected in layer 2. In this case, the nodes A, B, and C form a closed triangle in the multilayer network. A similar phenomenon can be observed for other triangles of the first type. In the second case, the sides of the triangle span three different layers. Taking nodes B, D, and E as an example, nodes B and D are connected at layer 1 but not at other layers, nodes B and E are connected at layer 2 but not at other layers, and nodes D and E are connected at layer 3 but not at other layers. In this case, the nodes B, D, and E form a closed triangle in the multilayer network. A similar phenomenon can be observed for other triangles of the second type.

However, the existence of a multilayer network greatly enriches the way triangles are formed in the network. The edges of a closed triangle may all be distributed in a certain layer or may be separately distributed in different layers. As shown in Figure 2, a three-layer network model is taken as an example to introduce two ways of forming a locally closed triangle across layers in a multilayer network. From this figure, we can obtain two kinds of triangles, corresponding to the two rows on the right of Figure 2. The sides of the first type of triangle are made up of two layers, while the sides of the second type of triangle span three different layers. Thus, it can be seen that the triangles in a multilayer network are greatly different from those in a single-layer network. The interaction between the various layers of the system in terms of clustering should be taken into account.

Many scholars have tried to solve this problem [29,30,31]. By extending the clustering coefficient in a multilayer brain network, we defined the cross-frequency clustering coefficient [32] as follows:

(2) $$C_{i}=\frac{\sum_{\alpha }\sum_{\alpha^{\prime }\neq \alpha }\sum_{\alpha^{\prime \prime }}\sum_{j\neq j,m\neq i}(a_{ij}^{\left[\alpha \right]}a_{jm}^{\left[\alpha^{\prime }\right]}a_{mi}^{\left[\alpha^{\prime \prime }\right]})}{\sum_{\alpha }\sum_{\alpha^{\prime }\neq \alpha }\sum_{j\neq i,m\neq i}(a_{ij}^{\left[\alpha \right]}a_{mi}^{\left[\alpha^{\prime }\right]})},$$

where $a_{ij}^{\left[\alpha \right]}=1$ if node i and node j are connected through a link on layer α. This formula represents the ratio of the number of closed triples formed by node *i* across layers to the number of connected triples formed across layers in a multilayer network [32]. It reflects the trend that nodes form locally connected triangles across different layers and is a measure of local information processing capacity in multilayer networks.

The multilayer clustering coefficient (MCC) of the entire network can be expressed as the average value of the MCC of all nodes and is defined as follows:

(3) $$C=\frac{1}{N}\sum_{i=1}^{N}C_{i},$$

### 2.8. Multilayer Network Integration

Similar to the degree index in the single-layer network, the degree value in the multilayer network is also a reflection of the importance of nodes. The degree value of nodes in the whole multilayer network can be obtained by summing the degree values of nodes in different layers, which is called the multilayer overlapping degree of nodes. Therefore, in a multilayer network, there may be a situation where the multilayer overlapping degree of node i and node j is the same, but they have different degree distributions at different layers. Thus, these two nodes play different roles in a multilayer network. The entropy of the multiplex degree (EMD) index can well reflect the distribution of nodes’ links in different layers to judge the specific functional roles of nodes in the whole multilayer network.

**Figure 3:**
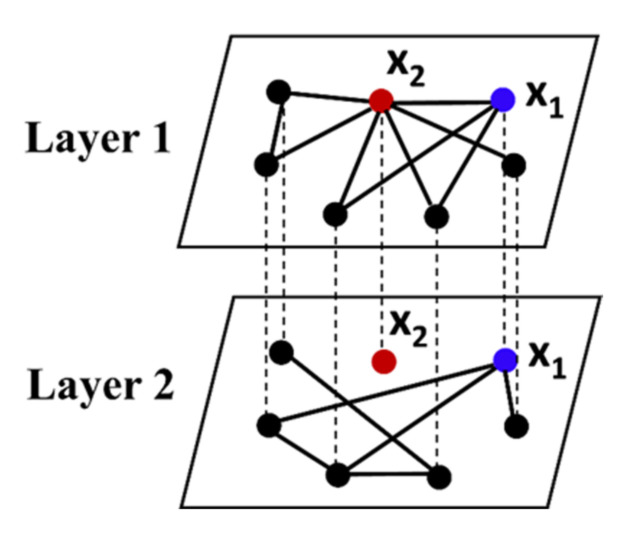
Diagram of the entropy of the multiplex degree. Node $x_{1}$ and node $x_{2}$ in the network correspond to a class of nodes with maximum and minimum entropy of the multiplex degree.

As shown in Figure 3, the physical meaning of the EMD index is demonstrated in a two-layer network. Take two nodes as an example; the $x_{1}\;$ and $x_{2}$ nodes have the same multilayer overlapping degree value. As for the $x_{1}\;$ node, it has the same degree distribution at each layer of the multilayer network, so it can spread the information acquired in each layer evenly among different layers. It can be regarded as the key node in the information exchange between layers, and its corresponding EMD value is the largest. However, for $x_{2}$ node, its network connection edges are all concentrated in the first layer of the multilayer network, while in the second layer, it belongs to the isolated node. Although it is a key node of information transmission in the first layer, it cannot transfer the information of the first layer to other layers, so it plays a certain blocking role in the inter-layer interaction. This node exists as a peripheral node in the whole multilayer network, and its corresponding EMD value is the minimum.

Therefore, the higher the EMD is, the more evenly the node’s links are distributed at each layer, and the more important the node is in the interlayer interaction. Therefore, EMD is a measure of global information processing capacity in a multilayer network. For a multilayer brain network, we defined the cross-frequency EMD [33] as follows:

(4) $$E_{i}=-\sum_{\alpha =1}^{M}\frac{K_{i}^{\left[\alpha \right]}}{O_{i}}\ln(\frac{K_{i}^{\left[\alpha \right]}}{O_{i}}),$$

where α  represents a layer in a multilayer network, *M* is the number of layers, $K_{i}^{\left[\alpha \right]}\;$ represents the degree value of node i on the α layer, and $O_{i}\;$ represents the overlapping degree of node *i* [33]. The relationship between $O_{i}\;$ and $K_{i}^{\left[\alpha \right]}$ is as follows:

(5) $$O_{i}=\sum_{\alpha =1}^{M}K_{i}^{\left[\alpha \right]},$$

The EMD of the whole network can be expressed as the average value of the EMD of all nodes:

(6) $$E=\frac{1}{N}\sum_{i=1}^{N}E_{i},$$

### 2.9. Parcellation into RSNs

Each of the brain regions divided using AAL was associated with its corresponding RSN. Five empirical functional networks could be extracted from the network templates [34,35]: the default mode network (DMN), attention network, sensorimotor network, subcortical network, and visual network (see Supplementary Material Table S1). By computing multilayer network metrics at the RSN level, the differences in functional subnetworks between normal control and patients with schizophrenia can be quantified.

### 2.10. Statistical Analysis

The distribution of data was checked before the statistical test method was selected. The two sets of data in this paper all obeyed a normal distribution and satisfied the same variance. Therefore, the independent-samples *t*-test was used to analyze the network indicators difference between groups (TD and SCHZ), and the significance level was set at 0.05. To control the false positive rate, we used false discovery rate (FDR) correction for the results of the statistical tests [36]. *P*-values were adjusted using the FDR correction by the R package ‘fdrtool’. The program is freely available from the Comprehensive R Archive Network (http://cran.r-project.org/ (accessed on 2nd November 2021)). As a result of the correction, the statistical significance level was *p* < 0.05. In addition, the nonparametric Spearman correlation coefficient R was used to test whether multilayer network indicators were related to the symptom scores (SANS and SAPS). Meanwhile, the clustering coefficient correlations between pairs of layers in normal controls and schizophrenia patients were computed by Spearman correlation coefficient R. The independent-samples *t*-test and the nonparametric Spearman correlation were obtained using SPSS 20.0 software.

## 3. Results

### 3.1. Network Integration and Segregation

**Figure 4:**
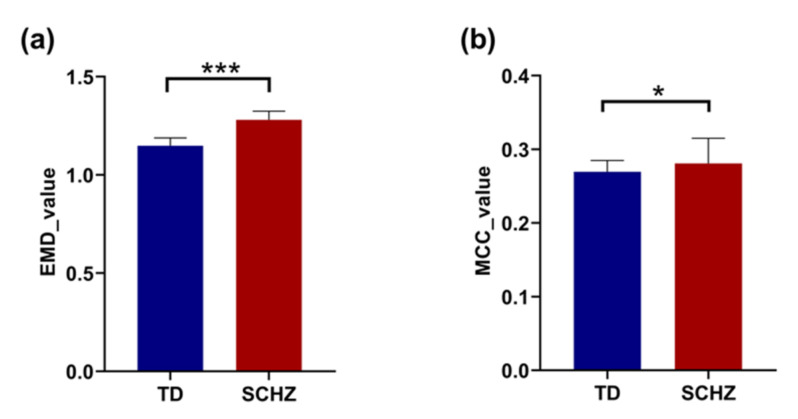
Panels (**a**,**b**) represent the differences in the entropy of the multiplex degree (EMD) and multilayer clustering coefficient (MCC) at the whole-brain level between control subjects and patients with schizophrenia (SCHZ), respectively. * indicates 0.01 < *p* < 0.05, *** indicates *p* = 0.000.

The difference between the two groups in terms of integration using EMD and segregation using MCC was explored at the whole-brain level. As seen in Figure 4, the EMD value (t(90) = −17.059, *p* = 0.000) and MCC value (t(90) = −2.179, *p* = 0.033) in schizophrenia were significantly higher than those of the control group.

**Figure 5:**
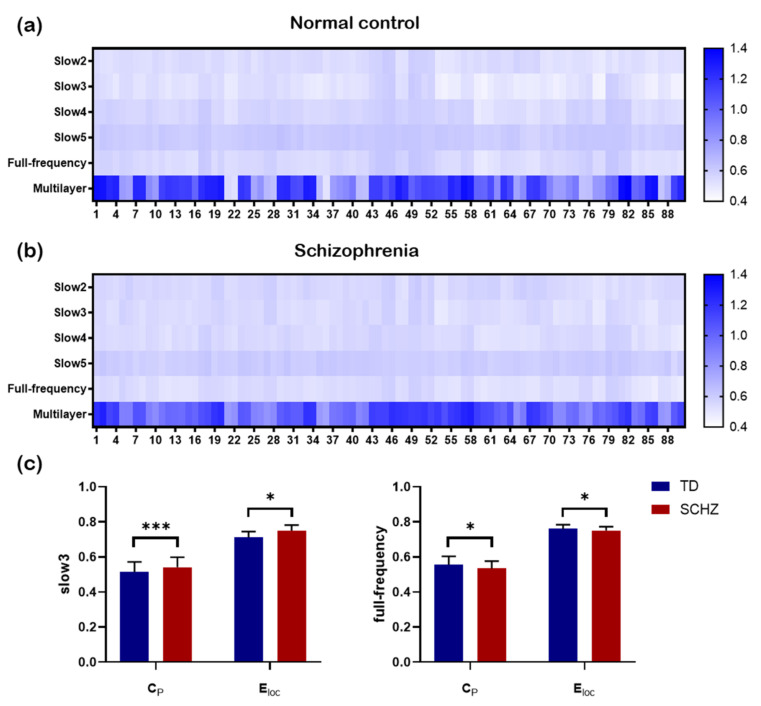
The clustering coefficient values of 90 brain regions. (**a**) The clustering coefficient for each region for four single-layer frequency-specific networks and a multilayer network in the healthy group is shown. (**b**) The clustering coefficient for each region for four single-layer frequency-specific networks and a multilayer network in patients with schizophrenia is depicted. (**c**) The differences in the clustering coefficient (C_p_) and local network efficiency (E_loc_) between the two groups in the slow3 frequency and full-frequency are shown. * indicates 0.01 < *p* < 0.05. *** indicates *p* = 0.000.

**Figure 6:**
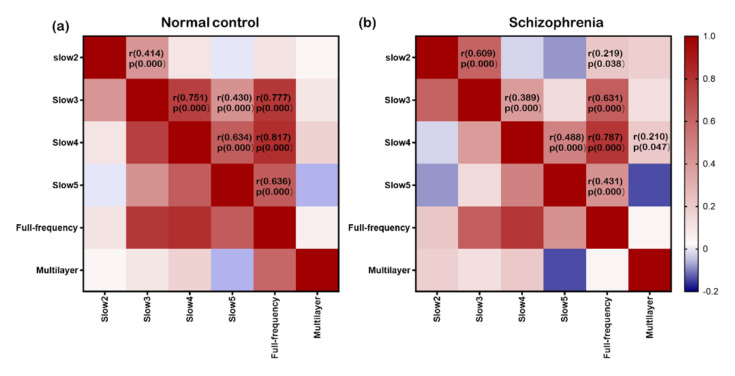
Panels (**a**,**b**) represent the clustering coefficient correlations between pairs of layers in normal controls and patients with schizophrenia. The self-correlations are equal to 1.

Figure 5a,b show the clustering coefficient values of 90 brain regions of normal controls and patients with schizophrenia in the single-layer and multilayer networks, respectively. It can be seen that the clustering coefficient is very different in these two kinds of networks. Statistical analysis showed that there were significant differences in the clustering coefficient (C_p_) (slow3: t(90) = −2.323, *p* = 0.022; full-frequency: t(90) = 2.441, *p* = 0.016) and local network efficiency (E_loc_) (slow3: t(90) = −5.927, *p* = 0.000; full-frequency: t(90) = 2.009, *p* = 0.047) between the two groups in the slow3 frequency band and full-frequency band (Figure 5c). Furthermore, we analyzed the correlation between the cluster coefficient sequences of all brain regions in different networks (Figure 6).

### 3.2. RSNs Differences

**Figure 7:**
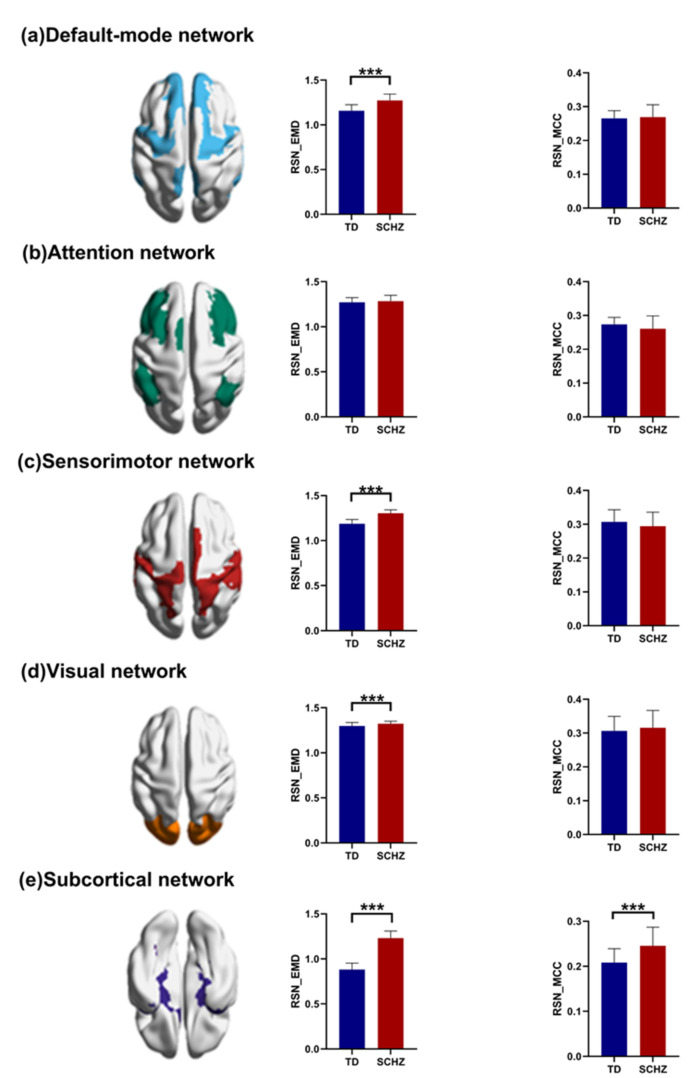
The differences in the entropy of the multiplex degree (EMD) and multilayer clustering coefficient (MCC) at the RSN level between controls and patients with schizophrenia (SCHZ). *** indicates *p* = 0.000.

The differences between the two groups at the RSN level are shown in Figure 7, and these results were adjusted by FDR. By comparing the average EMD and MCC, we found that the EMD was significantly different in the DMN (t(90) = −9.002, p(FDR) = 0.000), sensorimotor network (t(90) = −14.451, p(FDR) = 0.000), visual network (t(90) = −3.989, p(FDR) = 0.000), and subcortical network (t(90) = −25.364, p(FDR) = 0.000). Furthermore, the MCC was significantly different in the subcortical network (t(90) = −5.677, p(FDR) = 0.000). In addition, the differences between the two groups at the RSN level of single-layer networks are shown in Supplementary Material Tables S2 and S3, and these results were adjusted by FDR.

### 3.3. Node Vulnerability

**Figure 8:**
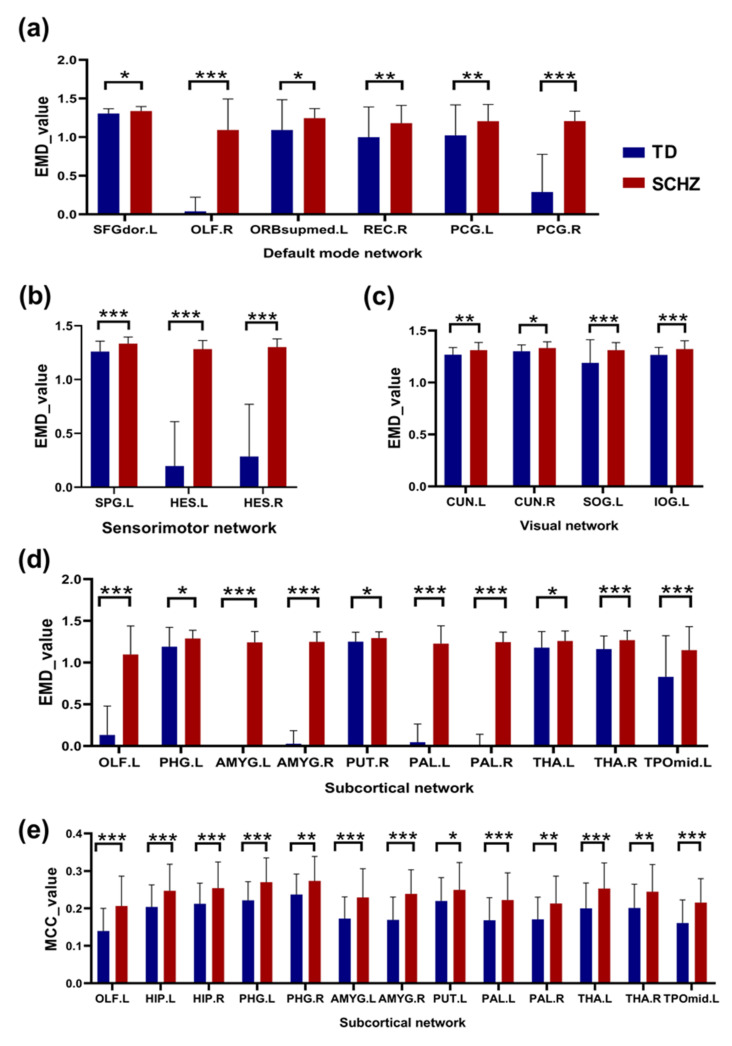
Differences between normal controls and patients with schizophrenia (SCHZ) in terms of node vulnerability. Panels (**a**–**d**) represent the damaged brain areas detected in the default mode network, sensorimotor network, visual network and subcortical network, respectively, of the schizophrenia patients according to the entropy of the multiplex degree (EMD). Panel (**e**) represents the damaged brain areas in the subcortical network of schizophrenia under the multilayer clustering coefficient index (MCC). Asterisks indicate the difference between the groups and were divided into three cases: * 0.01 < *p* < 0.05, ** 0.001 < *p* < 0.01, and *** *p* = 0.000.

**Figure 9:**
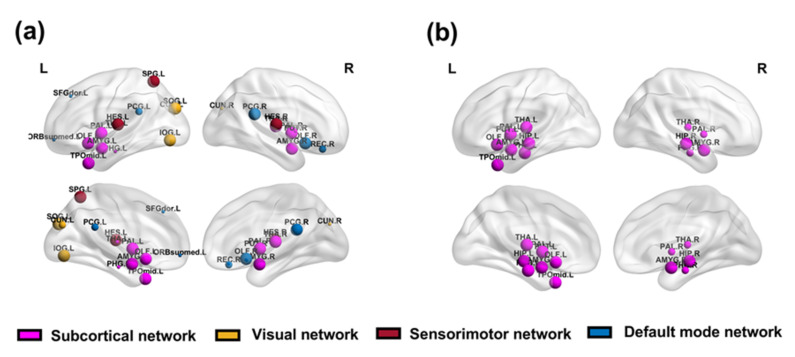
Cortical maps of damaged brain regions in schizophrenia. Panel (**a**) represents the cortical distribution of the damaged brain regions in the default mode network, sensorimotor network, visual network, and subcortical network of patients according to the entropy of the multiplex degree index. Panel (**b**) represents the cortical distribution of the damaged brain regions in the subcortical network of patients under the multilayer clustering coefficient index. The sizes of the nodes correspond to the significance of the difference between the groups. The smaller the *p*-value, the larger the node.

At the node level, the difference between the EMD and MCC groups was detected. We focused on the brain regions in the differential RSN mentioned in the previous section. For EMD (Table 2), there were a total of 23 abnormal brain regions in patients, among which 5 nodes belonged to the DMN (Figure 8a), 3 nodes belonged to the sensorimotor network (Figure 8b), 4 nodes belonged to the visual network (Figure 8c), and 11 nodes belonged to the subcortical network (Figure 8d). The cortical distribution of all of these brain regions is shown in Figure 9a. In the MCC index (Table 3), we found 13 abnormally damaged brain regions belonging to the subcortical network (Figure 8e), and these nodes are shown in Figure 9b. In addition, the differences between the two groups at the node level of single-layer networks were shown in Supplementary Material Tables S4 and S5, and these results were adjusted by FDR.

### 3.4. Correlation between Network Measures and Cognitive Scores

**Figure 10:**
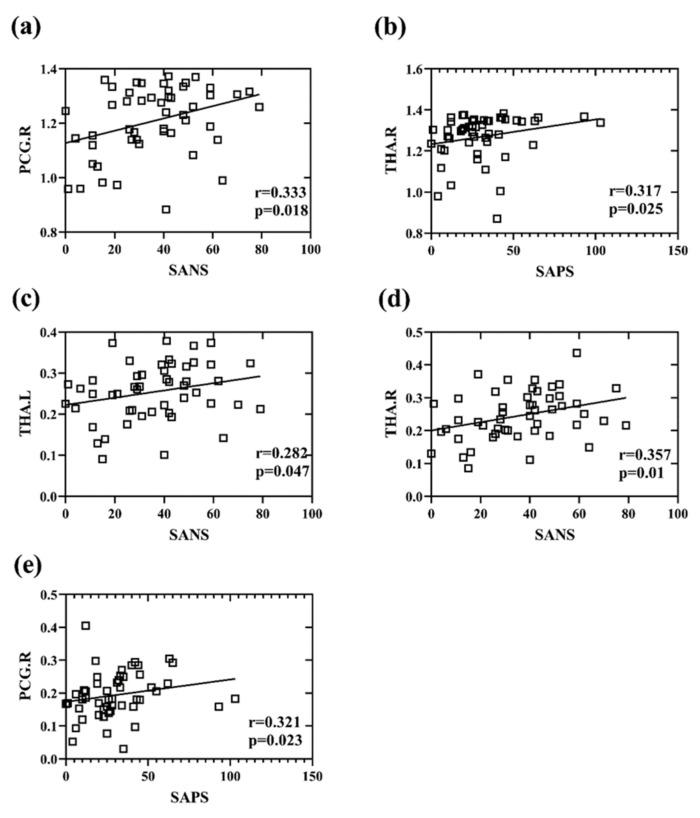
Graph (**a**) shows the correlation between the entropy of the multiplex degree of the right posterior cingulate gyrus (PCG.R) and the SANS. Graph (**b**) depicts the correlation between the entropy of the multiplex degree of the right thalamus (THA.R) and the SAPS. Graph (**c**) represents the correlation between the multilayer clustering coefficient of the left thalamus (THA.L) and the SANS. Graph (**d**) displays the correlation between the multilayer clustering coefficient of the THA.R and the SANS. Graph (**e**) demonstrates the correlation between the multilayer clustering coefficient of the PCG.R and the SAPS.

The correlation between damaged brain regions and positive symptom scores and between damaged brain regions and negative symptom scores of patients with schizophrenia was explored (Figure 10). The results showed that, for the EMD index, the right posterior cingulate gyrus (PCG.R) (r = 0.333, *p* = 0.018) was significantly correlated with SANS, and the right thalamus (THA.R) (r = 0.317, *p* = 0.025) was significantly correlated with SAPS. For the MCC index, the left thalamus (THA.L) (r = 0.282, *p* = 0.047) and THA.R (r = 0.357, *p* = 0.011) were significantly correlated with SANS, and the PCG.R (r = 0.321, *p* = 0.023) was significantly correlated with SAPS.

## 4. Discussion

In this study, we explored the dynamic topology characteristics of a multilayer network based on resting-state fMRI data. The mechanism of integration and separation of the multilayer brain network in patients with schizophrenia was studied. The results showed that both local and global information processing abilities were abnormally higher in the multilayer frequency brain network of patients with schizophrenia than in the control group and that there was a significant correlation between the impaired nodes and the cognitive scores of the patients.

### 4.1. High Integration and Segregation in the Multilayer Network of Patients with Schizophrenia

This study shows an increase in integration ability in the multilayer network of patients with schizophrenia, indicating that the interlayer information interaction ability had an increasing trend. One reason for this may be that the brain activity of patients is unstable. The results of a study on brain network reconstruction in patients with schizophrenia during n-back tasks showed that patients had the highest overall brain flexibility during working memory tasks, that the brain flexibility of their relatives was in the middle, and that healthy controls had the lowest flexibility [37]. In addition, a study based on resting-state fMRI data has consistently shown that the brains of patients are indeed significantly more flexible than those of healthy people [38]. This may represent a compensatory measure of brain dynamics, with frequent interactions between different systems even at rest.

Similarly, the segregation degree in the multilayer network of schizophrenia also showed an increasing trend, indicating that the local information processing ability across the frequency band was enhanced. In addition, this study also found that the local information-processing ability of the slow3 frequency band and full-frequency band were impaired. One possible explanation for our results is that cross-layer communication may have served as a compensatory mechanism in maintaining basic cognitive processes. For example, we found that intra-frequency C_p_ of pallidum (Pallidum.R) decreased for full-frequency single network and that cross-frequency EMD increased.

### 4.2. Aberrant RSNs in Patients with Schizophrenia

The results showed that the EMD of the DMN, sensorimotor network, and visual network in patients with schizophrenia is significantly higher than that of normal people. It indicates that these RSNs contribute the most to the enhancement of the global information processing ability. The functional network differences in the brains of patients have been reported in previous studies [39,40]. The distribution of connections within and between functional modules in patients changed, indicating the existence of unbalanced mechanisms of functional separation and integration.

The study found that the connection between functional networks especially in the DMN was reduced in patients with early schizophrenia [41]. It has also been reported as the strongest contributor to the structural differences in communities [42]. Most importantly, this dysfunction of functional connectivity in DMN is associated with positive symptoms in clinical symptoms, including delusions and hallucinations [43]. Therefore, schizophrenia is related to changes in the time frequency and spatial location of the DMN. A study based on fMRI has detected abnormal functional connections in the sensorimotor network at low frequencies (0.01–0.08 Hz) in schizophrenia, and the functional connections between this region and the patient’s visual region also tend to be weakened [44]. These findings highlight the impairment in information integration between the sensory and perceptual systems of patients with schizophrenia and provide new neurological evidence for the hypothesis of perceptual and cognitive dysfunction in patients with schizophrenia. Taken together, findings from previous studies and our results suggest that the brain networks of patients with schizophrenia may be altered.

Among all brain regions, those with significant differences in the DMN include the left dorsal superior frontal gyrus (SFGdor.L), right olfactory cortex (OLF.R), left medial orbital superior frontal gyrus (ORBsupmed.L), right gyrus rectus (REC.R), and posterior cingulate gyrus (PCG.L/R). Brain regions with significant differences in the sensorimotor network included the left superior parietal gyrus (SPG.L) and Heschl gyrus (HES.L/R). Brain regions with significant differences in the visual network include the cuneus (CUN.L/R), left superior occipital gyrus (SOG.L), and left inferior occipital gyrus (IOG.L). In addition, the PCG.R was associated with the SANS of the patients according to the EMD index. The PCG.R was associated with the SAPS of the patients according to the MCC index.

Research has shown that the central executive network, dominated by the dorsolateral prefrontal cortex and lateral posterior parietal cortex, is disrupted in schizophrenia and that the loss of working memory in patients is at least partially attributable to specific pathological changes in the neuronal circuits of the dorsolateral prefrontal cortex [45]. The posterior cingulate gyrus, the core node of the DMN, has also been reported to be associated with schizophrenia [46]. The brain function of the orbitofrontal region mainly includes the processes of scenario processing, motivation processing, expectation processing and risk decision making. A previous study has also found abnormal brain blood flow and brain function in the orbitofrontal lobe of patients with schizophrenia, suggesting a possible correlation with negative symptoms in these patients [47]. In addition, the detection results of brain networks in patients showed that the length of the shortest path in the olfactory cortex, left middle occipital gyrus, and left superior occipital gyrus was significantly higher than that in normal controls, indicating impaired global information integration in these regions [48]. For these brain regions with significantly increased entropy values, we found that the frontal lobe, temporal lobe, occipital lobe, and basal lobe were predominant. Among them, the frontal, temporal, and occipital lobes have been shown to have more dysfunctional central nodes and reduced overall efficiency in patients with schizophrenia [49,50]. The frontal lobe, basal ganglia, and temporal lobes are thought to be the sites responsible for schizophrenia pathology [51].

### 4.3. Active Subcortical Network in Patients with Schizophrenia

The patients’ subcortical network had significantly enhanced global and local information-processing capacity in the multilayer network, indicating that the network made the greatest contribution to the cross-layer compensation mechanism caused by the disorder of intralayer information integration. The subcortical structure includes the basal ganglia and part of the limbic system regions, which are associated with various cognitive processes, such as memory, learning, attention, and emotion. Changes in this region may be related to clinical symptoms, behavioral abnormalities, and developmental abnormalities in patients. Subcortical structures have been shown to play an important role in higher-order cognitive functions such as attention, learning, motor control, and working memory; moreover, patients with schizophrenia tend to be deficient in these functions. Therefore, the study of subcortical structures is of great significance. The subcortical network shows intergroup differences under the two indexes of the EMD and MCC, and the brain regions mainly involved include the left and right hippocampus (HIP.L/R), left and right parahippocampal gyrus (PHG. L/R), left and right amygdala (AMYG.L/R), left and right putamen (PUT.L/R), left and right pallidum (PAL.L/R), left and right thalamus (THA.L/R), and left temporal pole: middle temporal gyrus (TPOmid.L). In addition, the THA.R was associated with the SAPS of the patients according to the EMD index. The THA.L/R was associated with the SANS of the patients according to the MCC index.

A previous study conducted MRI scanning analysis on the brains of 2028 patients with schizophrenia and found that the brain volume of the subcortical network of the patients was significantly different from that of people with normal brain function, mainly evident in the small volumes of the hippocampus, amygdala, thalamus, and intracranial and in the large volumes of the putamen, pallidum, and lateral ventricle of the patients [52]. Studies of adolescents with subclinical psychotic experiences also yielded consistent results for subcortical structures, demonstrating abnormal volume and laterality in the hippocampus, caudate nucleus, amygdala, and pallidum regions. This study suggests that there is already abnormality in the pallidum volume in early adolescence, which may be a precursor to psychosis [53].

### 4.4. Methodological Considerations and Limitations

Although multilayer networks can integrate the information from within and between layers at the same time, methods for the allocation of the weight of interlayer connections are not yet mature enough for this purpose. The physiological significance of the connections between different functional layers is less clear. In this study, the coupling of the same brain region between different layers was only analyzed, and the more complex cross-frequency coupling was not explored; this exploration will be a key research direction in the future.

The results of this study are based on a small data sample, which may lead to the lack of universality of the results. This method will be applied to larger data sets in future work. Like most studies of brain functional networks based on resting-state fMRI, the effects of physiological noise cannot be eliminated. Although we performed realignment in the data preprocessing procedure, it is still possible that some results may have been affected by motion artifacts. Due to the limitation of data sources, we have no way to obtain and eliminate physiological signals such as subjects’ respiration and heartbeat. Although covariate regression was carried out in the data preprocessing stage, it may still have had some influence.

## 5. Conclusions

This study constructs multilayer brain networks in patients with schizophrenia and healthy subjects from the multifrequency dimension, and the mechanism of functional integration and separation in the multilayer network was researched. The results showed that the trends of information interaction (integration mechanism) and cross-layer formation of local triangles (separation mechanism) in the multilayer frequency brain network of patients with schizophrenia were significantly enhanced compared with those of healthy controls. This change is focused on the DMN, sensorimotor network, subcortical network, and visual network. It is worth noting that the subcortical network is the most active functional network in the multilayer network of patients with schizophrenia; this activity is significantly higher in patients with schizophrenia than that of people with normal brain function according to the two indicators evaluated in this study. In addition, we found typically damaged brain regions, such as the thalamus and posterior cingulate gyrus, which were significantly associated with the clinical presentation of patients with schizophrenia. These results may serve as a possible com mechanism for intralayer dysfunction and may provide a new explanation for the neuropathologic mechanism in schizophrenia.

## Figures and Tables

**Table 1:** Subjects’ characteristics and symptom scores.

Group	TD	SCHZ	*p*-Value
Number of subjects	69	50	--
Age (mean ± SD)	31.83 ± 8.73	33.84 ± 6.51	0.156 ^1^
Sex (M/F)	42/27	38/12	0.083 ^2^
SAPS (mean ± SD)	--	30.92 ± 21.04	--
SANS (mean ± SD)	--	35.9 ± 19.21	--

Abbreviations: TD, typical development; SCHZ, schizophrenia; SD, standard deviation; SAPS, positive symptom scores; SANS, negative symptom scores; ^1^ Independent-samples *t*-test. ^2^ Pearson chi-square two-tailed test.

**Table 2:** Brain regions showing significant differences in the entropy of the multiplex degree.

ROI	Name	Abbreviation	Network	P (FDR)	SCHZ (SD)	TD (SD)
3	Frontal_Sup_L	SFGdor.L	Default mode	0.020	1.338 (0.058)	1.305 (0.061)
21	Olfactory_L	OLF.L	Subcortical	0.000	1.098 (0.338)	0.132 (0.344)
22	Olfactory_R	OLF.R	Subcortical	0.000	1.091 (0.398)	0.038 (0.182)
25	Frontal_Mid_Orb_L	ORBsupmed.L	Default mode	0.014	1.245 (0.122)	1.092 (0.388)
28	Rectus_R	REC.R	Default mode	0.009	1.180 (0.228)	0.999 (0.387)
35	Cingulum_Post_L	PCG.L	Default mode	0.009	1.205 (0.213)	1.021 (0.393)
36	Cingulum_Post_R	PCG.R	Default mode	0.000	1.208 (0.126)	0.289 (0.483)
39	ParaHippocampal_L	PHG.L	Subcortical	0.023	1.289 (0.098)	1.190 (0.229)
41	Amygdala_L	AMYG.L	Subcortical	0.000	1.242 (0.129)	0.000 (0.000)
42	Amygdala_R	AMYG.R	Subcortical	0.000	1.249 (0.116)	0.026 (0.156)
45	Cuneus_L	CUN.L	Visual	0.005	1.311 (0.073)	1.268 (0.068)
46	Cuneus_R	CUN.R	Visual	0.023	1.332 (0.059)	1.300 (0.061)
49	Occipital_Sup_L	SOG.L	Visual	0.000	1.311 (0.071)	1.188 (0.221)
53	Occipital_Inf_L	IOG.L	Visual	0.000	1.322 (0.079)	1.265 (0.072)
59	Parietal_Sup_L	SPG.L	Sensorimotor	0.000	1.334 (0.060)	1.260 (0.095)
74	Putamen_R	PUT.R	Subcortical	0.043	1.293 (0.075)	1.250 (0.113)
75	Pallidum_L	PAL.L	Subcortical	0.000	1.227 (0.211)	0.045 (0.216)
76	Pallidum_R	PAL.R	Subcortical	0.000	1.245 (0.119)	0.015 (0.124)
77	Thalamus_L	THA.L	Subcortical	0.038	1.258 (0.118)	1.179 (0.191)
78	Thalamus_R	THA.R	Subcortical	0.000	1.269 (0.111)	1.163 (0.154)
79	Heschl_L	HES.L	Sensorimotor	0.000	1.282 (0.080)	0.196 (0.409)
80	Heschl_R	HES.R	Sensorimotor	0.000	1.302 (0.074)	0.285 (0.482)
87	Temporal_Pole_Mid_L	TPOmid.L	Subcortical	0.000	1.149 (0.278)	0.829 (0.489)

**Table 3:** Brain regions showing significant differences in the multilayer clustering coefficient.

ROI	Name	Abbreviation	Network	P (FDR)	SCHZ (SD)	TD (SD)
21	Olfactory_L	OLF.L	Subcortical	0.000	0.207 (0.079)	0.139(0.059)
37	Hippocampus_L	HIP.L	Subcortical	0.000	0.247 (0.071)	0.203 (0.058)
38	Hippocampus_R	HIP.R	Subcortical	0.000	0.254 (0.070)	0.212 (0.054)
39	Parahippocampal_L	PHG.L	Subcortical	0.000	0.270 (0.065)	0.221 (0.049)
40	Parahippocampal_R	PHG.R	Subcortical	0.004	0.274 (0.065)	0.236 (0.054)
41	Amygdala_L	AMYG.L	Subcortical	0.000	0.230 (0.076)	0.172 (0.057)
42	Amygdala_R	AMYG.R	Subcortical	0.000	0.240 (0.064)	0.169 (0.060)
73	Putamen_L	PUT.L	Subcortical	0.033	0.249 (0.073)	0.219 (0.062)
75	Pallidum_L	PAL.L	Subcortical	0.000	0.223 (0.073)	0.168 (0.059)
76	Pallidum_R	PAL.R	Subcortical	0.002	0.213 (0.073)	0.170 (0.058)
77	Thalamus_L	THA.L	Subcortical	0.000	0.254 (0.068)	0.199 (0.067)
78	Thalamus_R	THA.R	Subcortical	0.002	0.245 (0.073)	0.201 (0.063)
87	Temporal_Pole_Mid_L	TPOmid.L	Subcortical	0.000	0.216 (0.064)	0.160 (0.061)

## Data Availability

The data were provided by the University of California LA Consortium for Neuropsychiatric Phenomics study, which was approved by the UCLA Institutional Review Board.

## Supplementary Materials

The following supporting information can be downloaded at: https://www.mdpi.com/article/10.3390/brainsci12030368/s1, Table S1: Resting-state network assignments of automated anatomical labeling (AAL) template; Table S2: Resting-state networks (RSNs) showing significant differences in the clustering coefficient (C_p_); Table S3: Resting-state networks (RSNs) showing significant differences in the local network efficiency (E_loc_); Table S4: Brain regions showing significant differences in the clustering coefficient (C_p_); Table S5: Brain regions showing significant differences in the local network efficiency (E_loc_).
