# Supplementary material for: Functional Integration and Segregation in a Multilayer Network Model of Patients with Schizophrenia
Source: Brain Sci. 2022 Mar 10;12(3):368. doi: 10.3390/brainsci12030368 (PMC8946586; doi:10.3390/brainsci12030368)
Supplement: Supplementary file 1 [file brainsci-12-00368-s001.zip › brainsci-1599612-supplementary-done.pdf]

brain sciences

MDPI

**Table S1.** Resting-state network assignments of automated anatomical labeling (AAL) template.

| Name | Acronyms | RSN | Name | Acronyms | RSN |
|---|---|---|---|---|---|
| Precentral_L | PreCG.L | Sensorimo-or | Lingual_L | LING.L | Visual |
| Precentral_R | PreCG.R | Sensorimo-or | Lingual_R | LING.R | Visual |
| Frontal_Sup_L | SFGdor.L | Default node | Occipital_Sup_L | SOG.L | Visual |
| Frontal_Sup_R | SFGdor.R | Default node | Occipital_Sup_R | SOG.R | Visual |
| Frontal_Sup_Orb_L | ORBsup.L | Attention | Occipital_Mid_L | MOG.L | Visual |
| Frontal_Sup_Orb_R | ORBsup.R | Default node | Occipital_Mid_R | MOG.R | Visual |
| Frontal_Mid_L | MFG.L | Attention | Occipital_Inf_L | IOG.L | Visual |
| Frontal_Mid_R | MFG.R | Attention | Occipital_Inf_R | IOG.R | Visual |
| Frontal_Mid_Orb_L | ORBmid.L | Attention | Fusiform_L | FFG.L | Visual |
| Frontal_Mid_Orb_R | ORBmid.R | Attention | Fusiform_R | FFG.R | Visual |
| Frontal_Inf_Oper_L | IFGoperc.L | Attention | Postcentral_L | PoCG.L | Sensorimotor |
| Frontal_Inf_Oper_R | IFGoperc.R | Attention | Postcentral_R | PoCG.R | Sensorimotor |
| Frontal_Inf_Tri_L | IFGtriang.L | Attention | Parietal_Sup_L | SPG.L | Sensorimotor |
| Frontal_Inf_Tri_R | IFGtriang.R | Attention | Parietal_Sup_R | SPG.R | Sensorimotor |
| Frontal_Inf_Orb_L | ORBinf.L | Attention | Parietal_Inf_L | IPL.L | Attention |
| Frontal_Inf_Orb_R | ORBinf.R | Attention | Parietal_Inf_R | IPL.R | Attention |
| Rolandic_Oper_L | ROL.L | Sensorimo-or | SupraMarginal_L | SMG.L | Sensorimotor |
| Rolandic_Oper_R | ROL.R | Sensorimo-or | SupraMarginal_R | SMG.R | Sensorimotor |
| Supp_Mo-tor_Area_L | SMA.L | Attention | Angular_L | ANG.L | Attention |
| Supp_Mo-tor_Area_R | SMA.R | Sensorimo-or | Angular_R | ANG.R | Attention |
| Olfactory_L | OLF.L | Subcortical | Precuneus_L | PCUN.L | Default mode |
| Olfactory_R | OLF.R | Subcortical | Precuneus_R | PCUN.R | Default mode |
| Frontal_Sup_Me-dial_L | SFGmed.L | Default node | Paracentral_Lob-ule_L | PCL.L | Sensorimotor |
| Frontal_Sup_Me-dial_R | SFGmed.R | Default node | Paracentral_Lob-ule_R | PCL.R | Sensorimotor |
| Frontal_Mid_Orb_L | ORBsup-med.L | Default node | Caudate_L | CAU.L | Subcortical |
| Frontal_Mid_Orb_R | ORBsup-med.R | Default node | Caudate_R | CAU.R | Subcortical |
| Rectus_L | REC.L | Default node | Putamen_L | PUT.L | Subcortical |
| Rectus_R | REC.R | Default mode | Putamen_R | PUT.R | Subcortical |
| Insula_L | INS.L | Sensorimo-tor | Pallidum_L | PAL.L | Subcortical |
| Insula_R | INS.R | Sensorimo-tor | Pallidum_R | PAL.R | Subcortical |
| Cingulum_Ant_L | ACG.L | Default mode | Thalamus_L | THA.L | Subcortical |

| Name | Acronyms | RSN | Name | Acronyms | RSN |
|---|---|---|---|---|---|
| Cingulum_Ant_R | ACG.R | Default mode | Thalamus_R | THA.R | Subcortical |
| Cingulum_Mid_L | DCG.L | Subcortical | Heschl_L | HES.L | Sensorimotor |
| Cingulum_Mid_R | DCG.R | Subcortical | Heschl_R | HES.R | Sensorimotor |
| Cingulum_Post_L | PCG.L | Default mode | Temporal_Sup_L | STG.L | Sensorimotor |
| Cingulum_Post_R | PCG.R | Default mode | Temporal_Sup_R | STG.R | Sensorimotor |
| Hippocampus_L | HIP.L | Subcortical | Temporal_Pole_Sup_L | TPOsup.L | Attention |
| Hippocampus_R | HIP.R | Subcortical | Temporal_Pole_Sup_R | TPOsup.R | Sensorimotor |
| ParaHippocampal_L | PHG.L | Subcortical | Temporal_Mid_L | MTG.L | Default mode |
| ParaHippocampal_R | PHG.R | Subcortical | Temporal_Mid_R | MTG.R | Default mode |
| Amygdala_L | AMYG.L | Subcortical | Temporal_Pole_Mid_L | TPOmid.L | Subcortical |
| Amygdala_R | AMYG.R | Subcortical | Temporal_Pole_Mid_R | TPOmid.R | Subcortical |
| Calcarine_L | CAL.L | Visual | Temporal_Inf_L | ITG.L | Attention |
| Calcarine_R | CAL.R | Visual | Temporal_Inf_R | ITG.R | Default mode |
| Cuneus_L | CUN.L | Visual | | | |
| Cuneus_R | CUN.R | Visual | | | |

**Table S2.** Resting-state networks (RSNs) showing significant differences in the clustering coefficient ($C_P$)

| ROI | Name | Frequency band | P (FDR) | SCHZ (SD) | TD (SD) |
|---|---|---|---|---|---|
| 2 | Visual | Full-Frequency | 0.004 | 0.54(0.05) | 0.58(0.05) |
| 3 | Attention | Slow 3 | 0.036 | 0.54(0.07) | 0.50(0.07) |
| 4 | Default Mode | Slow 3 | 0.036 | 0.54(0.06) | 0.51(0.07) |
| 5 | Subcortical | Slow 3 | 0.014 | 0.53(0.05) | 0.49(0.06) |

**Table S3.** Resting-state networks (RSNs) showing significant differences in the local network efficiency ($E_{loc}$)

| ROI | Name | Frequency band | P (FDR) | SCHZ (SD) | TD (SD) |
|---|---|---|---|---|---|
| 2 | Visual | Full-Frequency | 0.011 | 0.76(0.03) | 0.89(0.03) |
| 1 | Sensorimotor | Slow 3 | 0.001 | 0.75(0.04) | 0.72(0.05) |
| 2 | Visual | Slow 3 | 0.004 | 0.75(0.02) | 0.73(0.03) |
| 3 | Attention | Slow 3 | 0.001 | 0.74(0.04) | 0.70(0.05) |
| 4 | Default Mode | Slow 3 | 0.001 | 0.75(0.03) | 0.71(0.04) |
| 5 | Subcortical | Slow 3 | 0.001 | 0.74(0.03) | 0.69(0.04) |

**Table S4.** Brain regions showing significant differences in the clustering coefficient ($C_P$)

| ROI | Name | Frequency band | Network | P (FDR) | SCHZ (SD) | TD (SD) |
|---|---|---|---|---|---|---|
| 17 | Rolandic_Oper_L | Full-Frequency | Sensorimotor | 0.001 | 0.55(0.05) | 0.61(0.07) |
| 18 | Rolandic_Oper_R | Full-Frequency | Sensorimotor | 0.031 | 0.56(0.06) | 0.60(0.07) |
| 43 | Calcarine_L | Full-Frequency | Visual | 0.031 | 0.54(0.07) | 0.59(0.08) |
| 44 | Calcarine_R | Full-Frequency | Visual | 0.001 | 0.55(0.07) | 0.62(0.08) |
| 45 | Cuneus_L | Full-Frequency | Visual | 0.040 | 0.58(0.07) | 0.62(0.08) |
| 47 | Lingual_L | Full-Frequency | Visual | 0.031 | 0.53(0.07) | 0.57(0.07) |
| 49 | Occipital_Sup_L | Full-Frequency | Visual | 0.041 | 0.53(0.07) | 0.57(0.07) |
| 50 | Occipital_Sup_R | Full-Frequency | Visual | 0.041 | 0.58(0.08) | 0.62(0.07) |
| 74 | Putamen_R | Full-Frequency | Subcortical | 0.031 | 0.52(0.06) | 0.56(0.08) |
| 76 | Pallidum_R | Full-Frequency | Subcortical | 0.031 | 0.56(0.06) | 0.60(0.07) |

| | | | | | | |
|---|---|---|---|---|---|---|
| 80 | Heschl_R | Full-Frequency | Sensorimotor | 0.031 | 0.58(0.06) | 0.62(0.07) |
| 82 | Temporal_Sup_R | Full-Frequency | Sensorimotor | 0.031 | 0.54(0.06) | 0.58(0.06) |
| 86 | Temporal_Mid_R | Full-Frequency | Default Mode | 0.031 | 0.46(0.08) | 0.52(0.10) |
| 55 | Fusiform_L | Slow 2 | Visual | 0.033 | 0.56(0.07) | 0.49(0.09) |
| 21 | Olfactory_L | Slow 3 | Subcortical | 0.042 | 0.54(0.10) | 0.48(0.13) |
| 39 | ParaHippocampal_L | Slow 3 | Subcortical | 0.038 | 0.56(0.07) | 0.51(0.09) |
| 40 | ParaHippocampal_R | Slow 3 | Subcortical | 0.026 | 0.55(0.08) | 0.50(0.08) |
| 42 | Amygdala_R | Slow 3 | Subcortical | 0.038 | 0.57(0.08) | 0.51(0.10) |
| 55 | Fusiform_L | Slow 3 | Visual | 0.042 | 0.52(0.08) | 0.47(0.10) |
| 59 | Parietal_Sup_L | Slow 3 | Sensorimotor | 0.006 | 0.55(0.12) | 0.46(0.12) |
| 60 | Parietal_Sup_R | Slow 3 | Sensorimotor | 0.003 | 0.54(0.11) | 0.44(0.13) |
| 61 | Parietal_Inf_L | Slow 3 | Attention | 0.042 | 0.54(0.11) | 0.470.11) |
| 67 | Precuneus_L | Slow 3 | Default Mode | 0.002 | 0.56(0.10) | 0.47(0.11) |
| 68 | Precuneus_R | Slow 3 | Default Mode | 0.001 | 0.57(0.09) | 0.47(0.10) |
| 89 | Temporal_Inf_L | Slow 3 | Attention | 0.003 | 0.53(0.08) | 0.45(0.11) |
| 90 | Temporal_Inf_R | Slow 3 | Default Mode | 0.003 | 0.52(0.07) | 0.45(0.11) |

**Table S5.** Brain regions showing significant differences in the local network efficiency ($E_{loc}$)

| ROI | Name | Frequency band | Network | P (FDR) | SCHZ (SD) | TD (SD) |
|---|---|---|---|---|---|---|
| 17 | Rolandic_Oper_L | Full-Frequency | Sensorimotor | 0.001 | 0.77(0.03) | 0.80(0.03) |
| 44 | Calcarine_R | Full-Frequency | Visual | 0.007 | 0.76(0.04) | 0.80(0.04) |
| 55 | Fusiform_L | Slow 2 | Visual | 0.015 | 0.76(0.04) | 0.71(0.07) |
| 4 | Frontal_Sup_R | Slow 3 | Default Mode | 0.047 | 0.73(0.08) | 0.069(0.11) |
| 5 | Frontal_Sup_Orb_L | Slow 3 | Attention | 0.049 | 0.77(0.07) | 0.74(0.09) |
| 7 | Frontal_Mid_L | Slow 3 | Attention | 0.026 | 0.75(0.10) | 0.70(0.08) |
| 12 | Frontal_Inf_Oper_R | Slow 3 | Attention | 0.014 | 0.74(0.07) | 0.59(0.11) |
| 14 | Frontal_Inf_Tri_R | Slow 3 | Attention | 0.030 | 0.75(0.07) | 0.71(0.09) |
| 15 | Frontal_Inf_Orb_L | Slow 3 | Attention | 0.034 | 0.74(0.06) | 0.70(0.09) |
| 16 | Frontal_Inf_Orb_R | Slow 3 | Attention | 0.018 | 0.75(0.06) | 0.71(0.09) |
| 19 | Supp_Motor_Area_L | Slow 3 | Attention | 0.049 | 0.76(0.09) | 0.71(0.12) |
| 21 | Olfactory_L | Slow 3 | Subcortical | 0.001 | 0.75(0.08) | 0.67(0.11) |
| 22 | Olfactory_R | Slow 3 | Subcortical | 0.001 | 0.74(0.07) | 0.68(0.09) |
| 24 | Frontal_Sup_Medial_R | Slow 3 | Default Mode | 0.049 | 0.77(0.05) | 0.74(0.07) |
| 27 | Rectus_L | Slow 3 | Default Mode | 0.049 | 0.78(0.06) | 0.75(0.10) |
| 28 | Rectus_R | Slow 3 | Default Mode | 0.025 | 0.78(0.06) | 0.74(0.08) |
| 33 | Cingulum_Mid_L | Slow 3 | Subcortical | 0.003 | 0.75(0.07) | 0.69(0.11) |
| 34 | Cingulum_Mid_R | Slow 3 | Subcortical | 0.003 | 0.75(0.08) | 0.68(0.10) |
| 35 | Cingulum_Post_L | Slow 3 | Default Mode | 0.008 | 0.73(0.08) | 0.67(0.10) |
| 38 | Hippocampus_R | Slow 3 | Subcortical | 0.003 | 0.76(0.06) | 0.70(0.9) |
| 39 | ParaHippocampal_L | Slow 3 | Subcortical | 0.001 | 0.78(0.04) | 0.72(0.8) |
| 40 | ParaHippocampal_R | Slow 3 | Subcortical | 0.002 | 0.77(0.05) | 0.72(0.9) |
| 42 | Amygdala_R | Slow 3 | Subcortical | 0.001 | 0.77(0.05) | 0.71(0.09) |
| 54 | Occipital_Inf_R | Slow 3 | Visual | 0.010 | 0.70(0.08) | 0.64(0.12) |
| 55 | Fusiform_L | Slow 3 | Visual | 0.001 | 0.75(0.06) | 0.69(0.10) |
| 56 | Fusiform_R | Slow 3 | Visual | 0.002 | 0.74(0.06) | 0.68(0.10) |
| 58 | Postcentral_R | Slow 3 | Sensorimotor | 0.034 | 0.76(0.07) | 0.72(0.11) |
| 59 | Parietal_Sup_L | Slow 3 | Sensorimotor | 0.001 | 0.75(0.09) | 0.66(0.10) |
| 60 | Parietal_Sup_R | Slow 3 | Sensorimotor | 0.001 | 0.74(0.08) | 0.64(0.11) |
| 61 | Parietal_Inf_L | Slow 3 | Attention | 0.004 | 0.75(0.08) | 0.68(0.11) |
| 64 | SupraMarginal_R | Slow 3 | Sensorimotor | 0.049 | 0.74(0.07) | 0.70(0.10) |
| 65 | Angular_L | Slow 3 | Attention | 0.001 | 0.74(0.06) | 0.67(0.10) |
| 66 | Angular_R | Slow 3 | Attention | 0.013 | 0.74(0.09) | 0.69(0.09) |
| 67 | Precuneus_L | Slow 3 | Default Mode | 0.000 | 0.77(0.06) | 0.68(0.10) |
| 68 | Precuneus_R | Slow 3 | Default Mode | 0.000 | 0.78(0.08) | 0.69(0.10) |

| ROI | Name | Frequency band | Network | P (FDR) | SCHZ (SD) | TD (SD) |
|---|---|---|---|---|---|---|
| 69 | Paracentral_Lobule_L | Slow 3 | Sensorimotor | 0.007 | 0.75(0.08) | 0.68(0.13) |
| 70 | Paracentral_Lobule_R | Slow 3 | Sensorimotor | 0.001 | 0.76(0.09) | 0.67(0.12) |
| 71 | Caudate_L | Slow 3 | Subcortical | 0.013 | 0.72(0.09) | 0.65(0.12) |
| 72 | Caudate_R | Slow 3 | Subcortical | 0.010 | 0.73(0.09) | 0.67(0.11) |
| 74 | Putamen_R | Slow 3 | Subcortical | 0.040 | 0.74(0.08) | 0.70(0.09) |
| 77 | Thalamus_R | Slow 3 | Subcortical | 0.015 | 0.70(0.10) | 0.63(0.12) |
| 78 | Heschl_L | Slow 3 | Subcortical | 0.002 | 0.70(0.10) | 0.62(0.07) |
| 83 | Temporal_Pole_Sup_L | Slow 3 | Attention | 0.034 | 0.75(0.10) | 0.71(0.08) |
| 84 | Temporal_Pole_Sup_R | Slow 3 | Sensorimotor | 0.032 | 0.73(0.11) | 0.69(0.08) |
| 85 | Temporal_Mid_L | Slow 3 | Default Mode | 0.032 | 0.71(0.11) | 0.66(0.07) |
| 86 | Temporal_Mid_R | Slow 3 | Default Mode | 0.016 | 0.71(0.11) | 0.65(0.08) |
| 87 | Temporal_Pole_Mid_L | Slow 3 | Subcortical | 0.018 | 0.75(0.11) | 0.69(0.09) |
| 88 | Temporal_Pole_Mid_R | Slow 3 | Subcortical | 0.008 | 0.74(0.11) | 0.68(0.08) |
| 89 | Temporal_Inf_L | Slow 3 | Subcortical | 0.001 | 0.74(0.11) | 0.65(0.06) |
| 90 | Temporal_Inf_R | Slow 3 | Default Mode | 0.001 | 0.75(0.12) | 0.75(0.5) |
